# Ovary unusual colonic lesion

**DOI:** 10.1002/jgh3.12584

**Published:** 2021-06-02

**Authors:** Sanjivan Mudaliar, John D Chetwood, Samer Ghattas, Kevin Choi, Arthur Kaffes, Ken Liu

**Affiliations:** ^1^ AW Morrow Gastroenterology and Liver Centre Royal Prince Alfred Hospital Sydney New South Wales Australia; ^2^ Central Clinical School Department, Sydney Medical School University of Sydney Sydney New South Wales Australia; ^3^ Department of Radiology Royal Prince Alfred Hospital Sydney New South Wales Australia

**Keywords:** colonoscopy: E01.370.372.250.250.200, endoscopy E01.370.388.250, ovary: A05.360.319.114.630

## Abstract

We describe an unusual endoscopic finding, caused by a dominant ovarian follicle compressing a low‐lying ascending colon just inferior to a patulous retroverted cecum. Endoscopically detected extra‐colonic lesions represent a diverse group of pathologies, and it is important the endoscopist has an appreciation of the varied number of benign and malignant causes—including those of gynecological origin.

## Graphical abstract

We describe an unusual endoscopic finding, caused by a dominant ovarian follicle compressing a low‐lying ascending colon just inferior to a patulous retroverted cecum. Endoscopically detected extra extra‐colonic lesions represent a diverse group of pathologies, and it is important the endoscopist has an appreciation of the varied number of benign and malignant causes – including those of gynaecological origin.

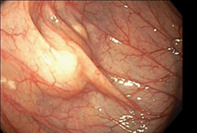

## Introduction

Though colonoscopy is more adept at ileocolonic mucosal evaluation, frequently the endoscopist may incidentally encounter submucosal, subserosal, or extra‐colonic lesions. These include benign lesions, such as colonic lipomas (with a reported incidence of 0.2–4.4%^1^), leiomyomas,^2^ lymphangiomas,^2^ pancreatic rests,^3^ compression from extracorporeal material^4^; as well as lesions that represent hazardous and malignant pathology such as gastrointestinal stromal tumors, neuroendocrine neoplasms, and extra‐colonic tumors.^3^, ^5^, ^6^ Thus, it is important to understand the significance and consider the further investigation of such incidental findings. We report an unusual case of a submucosal lesion found during colonoscopy, found to be a large, dominant ovarian follicle compressing a low‐lying ascending colon just inferior to a patulous retroverted cecum. The combination of the ovarian size and the anatomy likely contributed to this finding, and we are not aware that it has been previously reported in the medical literature.

## Case Report

**Figure 1:**
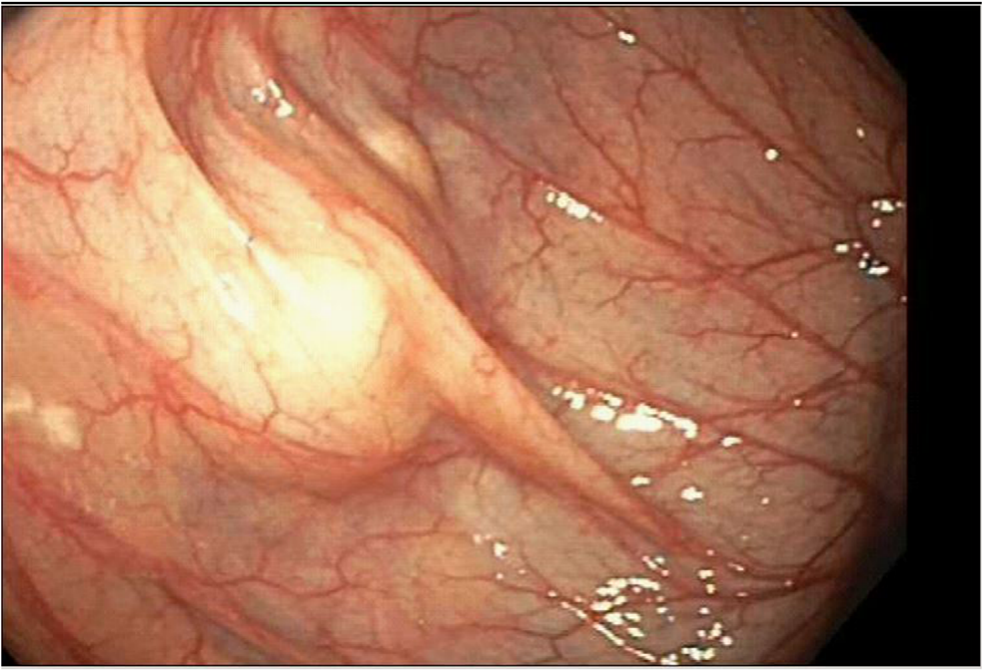
A submucosal lesion seen in the ascending colon.

**Figure 2:**
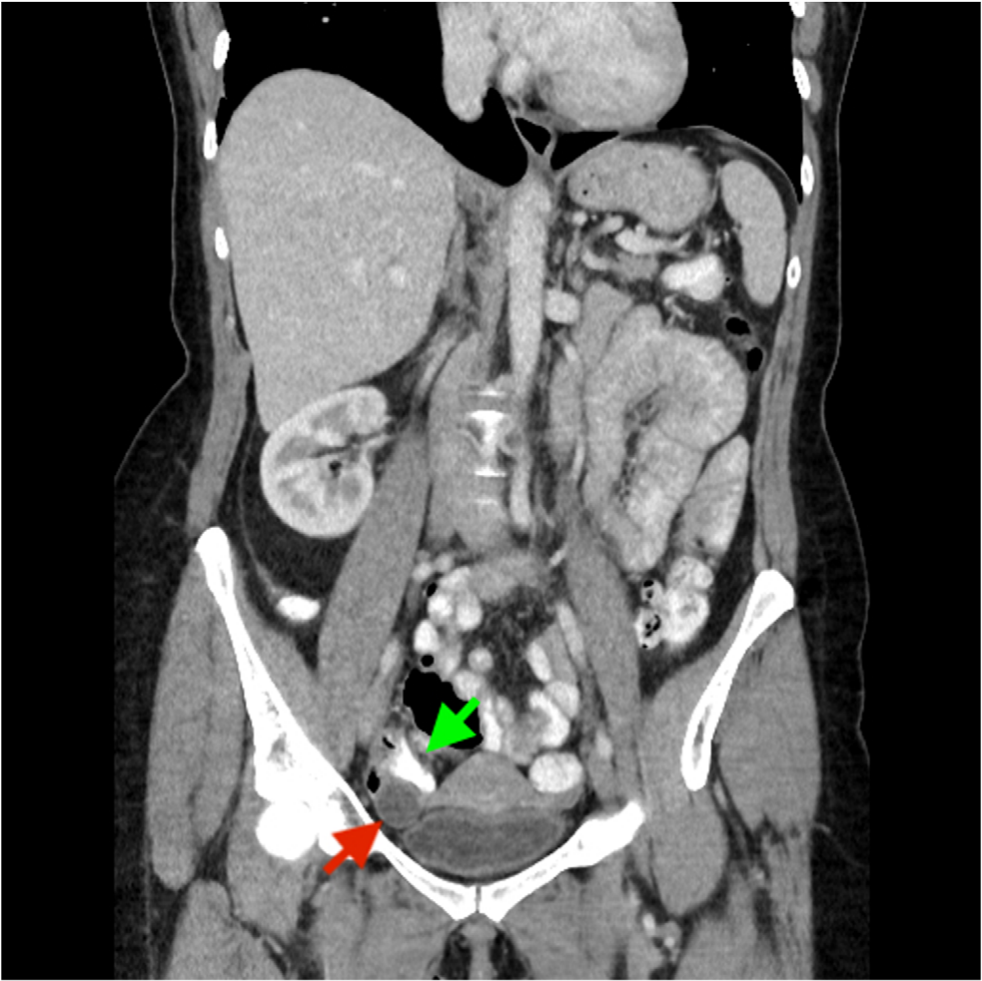
A 16 mm right ovarian dominant follicle (red arrow) seen on computed tomography compressing a low‐lying ascending colon (green arrow).

**Figure 3:**
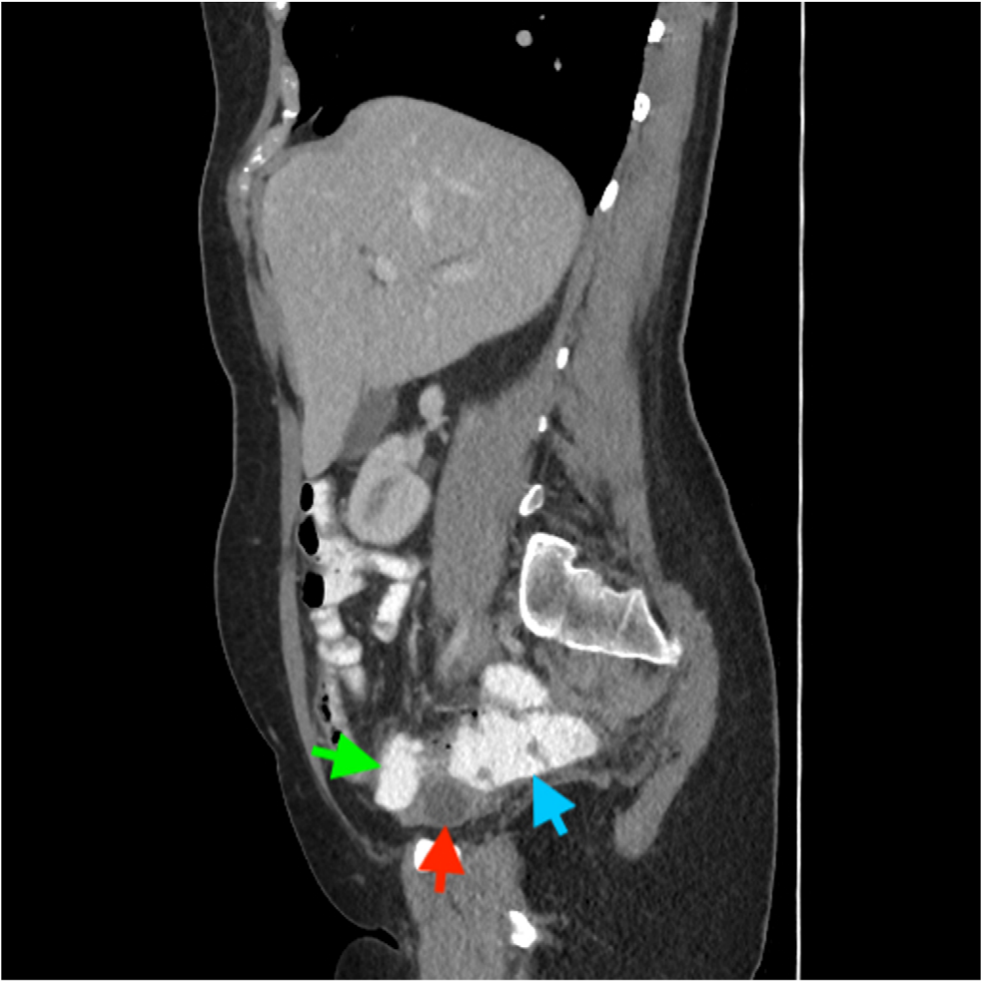
A 16 mm right ovarian dominant follicle (red arrow) seen on computed tomography compressing a low‐lying ascending colon (green arrow), just inferior to a patulous retroverted cecum (blue arrow).

A 51‐year‐old female was referred to the gastroenterology clinic for investigation of central abdominal pain, intermittent diarrhea, urgency, and rectal bleeding. Laboratory investigations including full blood count, iron studies, and C‐reactive protein levels were all within normal range. A colonoscopy arranged to further investigate her symptoms demonstrated internal hemorrhoids, simple sigmoid diverticulosis, and a 20 mm × 18 mm submucosal lesion in the proximal ascending colon (Fig. 1). The lesion did not indent when pushed with closed biopsy forceps (negative pillow sign). Subsequent computed tomography (CT) imaging of the abdominopelvic region (Figs 2, 3) found no colonic mural lesions but a 16 mm right ovarian dominant follicle (red arrow) compressing a low‐lying ascending colon (green arrow), just inferior to a patulous retroverted cecum (blue arrow). The patient was still having regular menses each month and was mid‐menstrual cycle at the time of her colonoscopy. The coincidental timing of her procedure with the development of a dominant right ovarian follicle resulted in indentation of the cecum seen on colonoscopy. After discussion at the multidisciplinary meeting, it was agreed that the endoscopic lesion could confidentially be accounted for by the benign ovarian pathology seen on CT—therefore no further intervention was deemed necessary.

## Discussion

The majority of submucosal lesions detected during endoscopy are small and incidental—however, they may also present with bleeding, obstruction, or metastases. Less than 15% of submucosal lesions are malignant but further investigation may be achieved with colonic endoscopic ultrasound (EUS) or cross‐sectional imaging. EUS also allows the evaluation of the histological layer, detection of associated lymphadenopathy, as well as biopsy—though cannot reliably visualize the muscularis mucosa within the mucosal layer.^3^

A dominant ovarian follicle is the follicle that is selected from the other tertiary follicles at the end of the luteal phase of a menstrual cycle. It can enlarge to 15–30 mm before release of the ovum, and several other gynecological pathologies may present as a colonic lesion including endometriosis, tubo‐ovarian abscess, and ovarian malignancies.^5^ The finding of a pathognomonic cushion or pillow sign (the indenting of the extra‐colonic lesion with biopsy forceps pressure) is purported to represent a soft lesion, thus reducing the chance of malignant origin—though such an approach may incorrectly classify softer malignant lesions as colonic mucosa associated lymphoid tissue (MALT) lymphomas.^7^ Further approaches used to classify submucosal lipomas include the naked fat sign (the endoscopic finding of extrusion of underlying yellowish fatty materials after repeated biopsies) and tent sign (rise of mucosa over the lipoma with the biopsy forceps).

We are not aware that a dominant ovarian follicle has previously been described to cause such an endoscopic appearance, and an endoscopist should be aware of such differentials when they encounter unusual submucosal colonic polypoid lesions—and consider further investigation to exclude malign causes.

## Consent

Written informed consent was obtained from the patient for the publication of their information and the associated images, and is presented anonymized. This conforms with the provisions of the Declaration of Helsinki, and thus formal ethical review was not required.

## References

[1] Vecchio R , Ferrara M , Mosca F , Ignoto A , Latteri F . Lipomas of the large bowel. Eur. J. Surg. 1996; 162: 915–19. 8956963

[2] Zuber M , Harder F . Benign tumors of the colon and rectum. In: Holzheimer RG , Mannick JA (eds). Surgical Treatment: Evidence‐Based and Problem‐Oriented. Munich: Zuckschwerdt, 2001. 21028753

[3] Standards of Practice Committee , Faulx AL , Kothari S *et al*. The role of endoscopy in subepithelial lesions of the GI tract. Gastrointest. Endosc. 2017; 85: 1117–32. 28385194 10.1016/j.gie.2017.02.022

[4] Eid JJ , Rodriguez A , Radecke JM , Murr MM . An unusual cecal mass on routine colonoscopy. J Surg Case Rep. 2014; 2014: rju119. 25389130 10.1093/jscr/rju119 PMC4226926

[5] Raś R , Barnaś E , Magierło JS *et al*. Preoperative colonoscopy in patients with a supposed primary ovarian cancer. Medicine. 2019; 98: e14929. 30896654 10.1097/MD.0000000000014929 PMC6709016

[6] Hellström M , Svensson MH , Lasson A . Extracolonic and incidental findings on CT colonography (virtual colonoscopy). AJR Am. J. Roentgenol. 2004; 182: 631–8. 14975961 10.2214/ajr.182.3.1820631

[7] Alvencar S , Holzwanger E , Dhingra R , Karagozian R , Olans L , Natov N . S1736 the pillow sign: is it always benign? Am. J. Gastroenterol. 2020; 115: S896. 10.14309/crj.0000000000000540 PMC7909236 33654703

